# Discussion on Intestinal Obstruction at the Bristol Meeting of the Bath and Bristol Branch of the British Medical Association

**Published:** 1885-03

**Authors:** 


THE BRISTOL

### Medico-Chirurgical Journal.

MARCH, 1885.

### DISCUSSION ON INTESTINAL OBSTRUCTION

*At the Bristol Meeting of the Bath and Bristol Branch of the British Medical Association, held Jany. 28th, 1885.*

---

Mr. Greig Smith: To-day it may truly be said that the abdominal cavity has passed from the hands of the pioneer into the grasp of the cultivator. The abdominal surgeon is no longer the mere explorer; he has many fruitful fields in full produce. But here and there, in the midst of this fertile tract, are patches of barren moorland; and, now that the bustle of discovery has slackened and he knows the ways of working, he sets himself to break up and cultivate these grounds also. His enterprise is one of the most hopeful. The surgery of the abdomen, from being the most unpromising field in our whole domain, we have seen become, in many respects, the most promising and successful; in many respects, but not in all. And most backward of all is the subject we are here to discuss this evening—that of Intestinal Obstruction. May we hope that for this also an improvement is near, and that our talk may do something to bring this improvement nearer?

I speak as if our advance in the treatment of this dire malady is to come from the surgeon; and herein, I am sure, all will agree with me. Physicians look to us to carry out this improvement; but we, on the other hand, look to physicians to guide and help us. When we have shown ourselves competent, promptly and with success, to deal with the condition, we may be sure that our friends will not be slow to give us the opportunity; on the other hand, we look to them to make this opportunity as favourable as they can by an accurate diagnosis, a judicious treatment, and a call to help that is not too late.

At the beginning of a discussion it is necessary to be accurate as to the terms we use. The name "Intestinal Obstruction" has already become (and this in itself is a hopeful sign of advance) too vague for us. It includes a range of diseases too wide, both as to pathology and as to treatment, for handy use. Not only does it include volvulus, intussusception, and that large class of strangulation by bands and through apertures—all herniæ, in fact; but it also takes cognizance of all blocks in the intestine from whatever cause arising, mechanical or vital, from outside or inside; and it includes even those local inflammations of the bowels, or their neighbours, which may by inflammatory paralysis or otherwise abrogate the contractile power of the intestines. Again, we speak of acute, sub-acute, and chronic obstruction; but this helps us but little. The symptom may be acute, while the cause may be chronic, and sub-acute symptoms may arise from a cause that is recent and acute. Or, thirdly, we may (like Mr. Bryant) notably circumscribe our meaning by the use of the term "Intestinal Strangulation;" and herein we derive a palpable advantage. If we can diagnose intestinal strangulation, we are certain that if the patient is to live it must be by operation. But there are other cases where strangulation of the bowel is not a necessary pathological result, and where death will certainly ensue if we do not operate. What are we to do here? It is on such points that your opinion is asked.

In deciding on a plan of treatment in any given case we are met with two considerations which are almost dilemmas.

First. Some cases of intestinal obstruction, acute as well as chronic, will right themselves without operation, and here delay is not harmful.

Second. Other cases will die without operation, and every hour spent in waiting diminishes the chances of operative success. Here delay is dangerous.

Now these are not equivalent propositions at the ends of a graduated scale, and it is impossible to strike a mean between them. To operate on all, or to leave all alone without operation, would be logically just as correct, and, practically, perhaps just as successful as to operate on a selected few that have been already tried with drugs. And this last is essentially all that the surgeon at present is asked to do, to try and remedy by operation what drugs have failed to do. Is this proceeding the one most likely to save the greatest number of patients? On the answer to this question must mainly depend the success of our treatment of these cases.

In our endeavours to get at an answer it will be well to limit the grounds of our enquiry. We may in the first place set aside those cases where an *accurate* diagnosis is possible. Such are stricture of the rectum, simple or malignant; the evident existence of growths or foreign bodies outside or inside the bowels. For these, rules of treatment are trustworthy and definite enough. Then we have a class of cases where a *probably accurate* diagnosis is possible. Such are chronic cases where there is a history pointing to some gradually narrowing stricture from growth or inflammation, or some other form of obstruction which is clearly cumulative. And, lastly, some cases of intussusception seen early can be diagnosed with something like certainty, and a good many more with a degree of high probability. All such cases we may put on one side.

Now, it happens that, in those cases where diagnosis is most trustworthy, medicinal treatment is most likely to be successful, and delay is least likely to be fatal. I was prepared, with diminished confidence, to make the same remarks of intussusception until I had read Mr. Treves' masterly summary in the *British Medical Journal* the other day, but now I cannot say so. And yet, calling to mind cases related to me by friends in practice, men who think and work a great deal, but publish nothing at all, I confess that I should be strongly disposed, in a marked case of intussusception seen early, to give a trial to distension by fluid. If this failed, I should operate at once; I should certainly not wait for the remote chance of exfoliation.

These are cases seen early, and diagnosed satisfactorily. There is another and important class of cases where accurate diagnosis is impossible, simply because the case has not been seen early enough and the abdomen is distended with gas. With the exception of stricture of the rectum, this class might contain examples of any of the others. What are we to do here? Reasoning the point out on paper, one would say operate. And yet within the last month I have three times deliberately advised departure from this conclusion. In one case (seen with Mr. Davies and Mr. Thomas, of Newport) age and probability of malignancy discountenanced operation; in another (with Mr. Hawkins, of this town), the same causes operated, with great stoutness as well; in a third (with Mr. Herapath, also of this town), the passage of flatus, and the history of a previous though slighter attack, made me, although the symptoms were very acute, hold my hand from day to day. The last patient recovered under belladonna;* the second died; and the first, when last heard of, was improving. In all these cases the gentlemen in charge had the same difficulties as I had.

With bowels less distended, these and other such cases might have been diagnosed with a high degree of probability; and all that can be said of them is, that each must be decided on its merits.

The most troublesome and the most dangerous class of all is where no diagnosis is possible beyond that of acute intestinal obstruction. A patient ordinarily in good health is suddenly seized with the well-known symptoms of the complaint, and we can diagnose no local cause. The cause may be of long standing; it may have been an affair of minutes. It may be a condition which medicine will alleviate, or even cure; or it may be one in which medicine is powerless. What are we to do?

Here we cannot have certainty; but we have probability, and on this probability we must act as if it were

* Since this was written this patient had a recurrence of the symptoms, and was successfully operated upon. A band from mesentery to bowel causing flexure by traction, was found to be the cause.

certainty. By far the greatest number of cases of acute obstruction with chronic causes have had previous symptoms of constipation or flatulence, or such; and by far the most numerous examples of acute obstruction are from recent causes: volvulus, or intussusception, or strangulation by bands. In all such cases we must, therefore, operate. In most obstruction is a sure forerunner of strangulation and death; in the others, done early, an operation may do no harm, and might be so managed as to do much good.

Recovery from any of these conditions is very rare, and an operation which promises any degree of success would command universal favour. Speaking for myself only, and looking back to cases and post-mortem examinations, my experience is little more than one long regret either that I had operated too late, or that I had not been permitted to operate at all. I can recall a case of death where the first incision through the abdominal wall showed a double strangulation by a Fallopian tube over an extra-uterine pregnancy; several cases of peritoneal bands, not so near the surface, but still easily discoverable; one of volvulus of the cæcum, and several cases of intussusception; all of which were permitted to die, while the removable cause was lying ready to our hands.

Was it necessary that all these cases should have been lost? Were the resources of our art too meagre to cope with them? Certainly they were not, and it was our own courage, diminished perhaps by our ignorance, that failed. Admit that we *may* save them, and I am sure we will show in time that we *can*.

Now, as to treatment—and first by medicines. We know we have got an obstruction to the passage of the intestinal contents, with its disastrous results treading close on its heels; and we know we may have a strangulation of the bowel as well.

If we are certain we have only obstruction, we may purge. But how often are we certain of this? The slightest doubt at the beginning positively forbids purging, and later on, when congestion appears, to be speedily followed by inflammation of the bowel, with ulceration perhaps, and impending perforation, purging is simply fatal. Purging, therefore, is a slender reed to lean upon, to be used only in the rarest instances, and even then with fear and trembling.

Our other resources in treatment have been presented in the formula, "rest, starvation and opium." I should like to see this formula changed into "rectal feeding and stimulation, a little morphia and much belladonna." It is needless to enlarge on the folly of attempting to administer anything whatever by the mouth: on this we are all agreed. But I think that the importance of sustaining the strength by stimulating and nourishing enemata has not been sufficiently insisted upon. Brandy above all, and in no stinted amount, I believe to be valuable. It diminishes shock, improves the pulse, relieves the griping pains, and I believe also diminishes the muscular irritability of the bowel. The universal recourse to a drop of neat brandy for a troublesome colic is a suggestive commentary on its undoubted value in these cases. As to the value of opium there can be no question. To begin with, a hypodermic injection of a third of a grain of morphia ameliorates some of the most pressing symptoms, but as a drug to use throughout the progress of the disease I believe it must surrender the palm to belladonna. A patient *looks* better under the tonic influence of belladonna; his colour is less blue, his aspect less cadaverous, and it has seemed to me that the local symptoms are not so pressing as under opium. On this point particularly I would ask for your opinion.

And now *as to the operation*. As to the *mode of it* our minds must be definitely and clearly made up. The patient is usually in such a condition that hesitation over any of the steps of the operation would be full of risk. We must know what we want to do; and do it promptly, decisively, and expeditiously.

Except in very rare instances, the median incision, below the umbilicus, is the one to be adopted. Through it only we can reach every part of the abdomen; and through this way the entrance can be made with most ease and expedition. I have once (in a case of Dr. Skinner's, of Clevedon) operated above the umbilicus, but the advantage was doubtful. The opening is made large enough to admit the hand into the abdomen.

When the opening is made the distended bowels, which lie on the surface, present at the opening. The hand is inserted, keeping these back, and carried first to the cæcum. If it is distended the obstruction is probably below it in the colon, and the hand carried superficially along the colon to the sigmoid flexure will probably alight on the cause of obstruction. If nothing is found, or if the cæcum is not distended, the hand is inserted under the distended coils from left to right, passing over the collapsed bowel if there be any, and the cause of obstruction felt for.

If nothing is now felt, I would then recommend a procedure which has been condemned. I would place over the opening a square yard or so of some layers of delicate linen or cotton material, or, better still, a large thin flat sponge, wrung out of warm carbolic lotion, and let the distended bowels come out under it. If they do not come out easily, I should coax them out, carefully covering them up as they extrude. The most distended coil, that next the obstruction, will probably come first, and the obstructing cause will in all likelihood be found at the root of it. I have done three operations since I decided on this course of procedure, and its success will certainly make me repeat it.

I cannot see the wisdom of passing along some dozen feet of bowel only to find one's self perhaps at the duodenum, with the necessity of going all the way backwards. Still less can I understand the necessity of exposing the bowel in inches till the obstruction is met with. The exposure over all is the same, and the manipulation is greater than if we let the bowel protrude as it will under its warmed and aseptic covering. Besides, the advantage of getting room to deal with the obstructing cause is no slight argument in favour of letting the bowels escape. And, finally, if there is any inflammation of the peritoneum, the manner in which the bowels stick to one's hand makes investigation difficult, and adds to the danger from traumatism. Those whose hands are frequently inside the abdominal cavity will, I am sure, appreciate what I say.

I can readily believe that the greatest mortality is found after exposure of the bowels. So has the greatest ovarian mortality been found after the longest incisions. But no one blames the incision. It is the sort of operation that necessitates the incision which kills, and not the incision itself. In the same way that an elongation of the incision in ovariotomy may diminish risk, so may an escape of the bowels properly guarded, by facilitating discovery and treatment of the cause of obstruction, help recovery.

And, further, this permitting of the escape of the bowels may have another advantage. As they lie on the abdomen, distended with fluid and gas, congested and perhaps inflamed, we can form an accurate opinion as to their chance of recovering themselves. The patient is probably under the influence of some sedative ; sickness has been less frequent, and there is probably great distension. As they lie, half-paralysed, their coils form acute flexures which almost block their lumina ; and even if we remove the immediate cause of obstruction they may not be able to pass on their contents. In such a condition I have made up my mind in future to evacuate by a small incision the contents of the bowel, and close the opening by suture.* Early operation would do away with such a necessity. But when shall we be able to operate early ?

Having decided as to the course to be pursued, medicinal or operative, it is my belief that we ought to adhere to this course throughout. From the beginning let it be drugs or knife. Let it never be that unfortunate compromise which is so frequently fatal—the knife when drugs fail. While we have been administering the drugs without success, the disease has been steadily killing the patient, and the knife finishes what the disease has already nearly completed.

It is all a question of diagnosis. If we could diagnose every case, I am sure we could save most. And why should we not diagnose every case ? When we see the marvellous exactitude with which a physician can diagnose obscure lesions of the heart or lung, we may surely hope that some day he may acquire the same mastery over the diagnosis of bowel complaints of this sort. The early symptoms have not been recorded with sufficient

* I have since done so, and successfully.

accuracy and detail, and their pathological meanings have not been properly worked out. To Mr. Treves, more than to anyone else, are we indebted for laborious and masterly efforts to help us here ; but his efforts have been sadly crippled by lax and inaccurate methods of reporting cases. Collective investigation alone will help us ; but not collective investigation after the superficial manner of our British Medical Association. If a score or so of our leading physicians were to meet a score of our leading surgeons, and, after thoroughly discussing the subject and deciding on important points to be observed and plans of treatment to be followed, to report their experiences in conclave at the end of two or three years, I am confident that we should be able to record an advance more decided than all the previous experiences of medicine have afforded. By any other plan we must wait for many years to get much more light.

To summarise what has been said :

1. Our treatment of intestinal obstruction varies according to the diagnosis.

2. Where diagnosis is certain we can at once decide on the plan of treatment, operative or medicinal, which promises most success.

3. Where diagnosis is uncertain and the symptoms are acute, operate at once.

4. Where diagnosis is doubtful or only probable, and the case is not extremely urgent, we may treat by drugs or operate, according as each case may warrant. The balance of favour ought to lean towards operation.

5. In all cases stimulating enemata are to be administered, and food by the mouth withheld.

6. Belladonna and opium are the only drugs deserving of confidence. One hypodermic injection of morphia, followed by repeated doses of belladonna, is probably the best combination of medicines.

In conclusion, if we were honestly to apply these doctrines to our individual cases, there would be much operating and little medicating. And is there cause for apprehension in this ? Medicine has done its best—and its worst. For its best, as for other small mercies, we are thankful; for its worst, the records are so terrible that if the procedure had been surgical, operators would have been forbidden the patient's bedside. Even in its present position, called in as a forlorn hope, or kept in the background as an untrustworthy and dangerous ally, surgery can show better records than medicine. But the surgery of the abdomen will not much longer consent to hold this position. Year by year, to its own glory and its clients' good, surgery has taken organ after organ from the domain of the physician and made it its own ; very soon will the most abdominal of all organs, the bowel, be the surgeon's too. I am assured that when we show ourselves worthy of the trust the physicians will be only too glad to let it abide with us. We cannot spring into the worthiness of trust all at once; experience can come only after trial. Therefore, I say, take us on probation. Let us have the part handling of these cases from the beginning ; let us watch them hourly from the moment of attack, ready to pounce on them at the fitting moment. For these cases are not to be entered as cases in the practitioner's list to be visited twice or thrice daily, but to be reckoned in the category of a parturient woman or a bleeding vessel, to be left only when the process is finished. In good faith and in full confidence I make this plea for surgery; and if you will trust it and encourage it, surgery will not fail you.

Mr. Treves (London): I may, in the first place, be allowed to congratulate Mr. Greig Smith upon his very able paper, and to express my entire concurrence with the chief statements he has enunciated. It would appear that the great difference of opinion that still exists as to the proper treatment of intestinal obstruction is, for the most part, a difference between physician and surgeon. The physician is disposed to temporize; to attach a supreme importance to the use of certain drugs, and even to cast a favourable eye upon certain somewhat vague methods, such as massage and the casual employment of electricity. He is not ashamed to confess that he is acting a little in the dark, and he holds a firm conviction that an operation should be a last resort. In this course of action, he entrenches himself behind the experience of "a case that got well without surgical treatment;" and it must be confessed that such cases are a sore let and hindrance to the surgeon. It is well known that patients presenting the gravest symptoms, and brought apparently near to the point of death, have recovered under the simplest treatment. That the pathology of these cases of recovery is a little obscure, the physician must acknowledge; that they are very rare, he must also own; but still the experience of one such is apt to influence his practice to a totally disproportionate extent through a long series of succeeding cases. On the other hand, the surgeon of the present day is a little too eager to use the knife on the slightest provocation. He is apt to regard laparotomy as a panacea for all abdominal ailments. "My man," he is inclined to say, "your bowels are not open; you are sick; your pulse is feeble; you must have your abdomen opened without delay." Abdominal surgery is, without doubt, making rapid progress; but in all vigorous scientific advances there is usually at first an element of exaggeration, a disposition to excess; and it is only with time that the proper mean is reached. Someone, imbued with the spirit of the Arabian Nights, has observed that there are men walking about in Germany without viscera, or with but a fractional part of the proper anatomical amount. Metres of their intestine have been resected; their spleens have been removed; one kidney has gone; they have neither pylorus nor gall bladder.

Certainly the physician has acted as a wholesome check in what may have been a too impetuous surgical career; and, so far as the patient is concerned, he has tempered the wind of progress to the shorn lamb. It is well, however, not to lose sight of the fact that the most startling of the recent methods adopted in abdominal surgery involve no new therapeutic principle; they embody no great revolutionary change in treatment. They consist merely in the tardy application to abdominal disease of principles in practice that have long been accepted so far as other parts of the body are concerned. The surgeon who resects a cancerous pylorus or a segment of strictured colon, or who cuts out an impacted foreign body or removes a degenerate kidney, is basing his procedure upon no novel data. His measures are certainly new in practice, but they are very old in principle; as old as the operation of excision for cancer of the lip, of lithotomy for stone, of amputation for destructive diseases in a limb.

If between physician and surgeon there is, in the matter of interference in some intestinal diseases, a great gulf fixed, it is a gulf that is largely occupied by bias. The commonest form of intestinal obstruction that the surgeon meets with is strangulated hernia, and this circumstance can hardly fail to prejudice him in favour of operation. The commonest form of obstruction that the physician meets with is that due to fæcal accumulation, and his experience of the treatment of that condition fosters a vigorous objection to the use of the knife in kindred disorders. Both practitioners will probably be agreed that to treat an acutely strangulated rupture with belladonna, electricity, and massage, is to do little more than encourage a therapeutical burlesque, while, on the other hand, operative interference in fæcal accumulation must rank as a surgical calamity.

It appears to me that one conspicuous and common fault in the treatment of intestinal obstruction that is advocated by some depends upon the custom of regarding intestinal obstruction as an individual disease. I take it that the term does not refer to a specific ailment, but is applied rather to a certain combination of symptoms. In a strict sense the term represents a clinical element that is common to many very varied and diverse pathological conditions. Those who propose a routine treatment for intestinal obstruction would be in a position to regard "dyspnœa" as a definite disease, and apply to it a definite and unvarying therapeutic measure. It has been recently urged, for example, that obstruction should be treated by rest, starvation, and opium; that this treatment should be methodical, should be adopted in all cases, and should be applied without reservation or compromise. Such advice is not in the direction of progress; it is a retrograde step—a lapse into the happy days when diagnosis was a dream, and when pathology was not.

If this be the counsel that we follow, then let us at once accept "dyspnœa" as a distinct disease, and "coated tongue" as a mortal malady; and let us treat every example of the one with the steam kettle, and every instance of the other with a rhubarb pill.

With regard to specific modes of treatment in acute cases, I can only endorse the excellent propositions of Mr. Greig Smith.

I cannot, however, agree with Mr. Smith in thinking that the best way to find the seat of obstruction is to allow the distended coils to escape from the abdomen. The condition of these coils is usually such as to forbid much handling; and I have been always disposed to think that in abdominal operations the less the exposure of peritoneal surfaces, the better are the prospects of the procedure.

More precise bases for active treatment will depend upon more accurate bases for diagnosis. And in this direction I think that more significance may be given to the character of the pain, to its persistence or intermittence, to its association with vomiting, and to its mode of onset and progress. More care, moreover, may well be bestowed upon the physical examination of the abdomen; upon the position and movements of distended coils; upon examinations through the rectal walls; upon auscultation of the colon, and upon careful and frequently repeated percussion. It is of interest to note that many surgeons still cling in a spirit of simple and trustful faith to the "long tube." They appear to regard it as a kind of fetish, as an inanimate thing to be invoked as a last resort, and when in dire distress. History tells us that this remarkable pipe has been passed many feet into the colon, that it has reached the transverse colon, and that it has even found its way into the cæcum. I can only say that the adventures of this tube and the anatomy of the colon do not quite agree; there is a discrepancy somewhere, and of course it may be that the bowel is at fault.

After many patient trials upon many dead bodies, I have not yet succeeded in passing this interesting pipe beyond the sigmoid flexure. Several feet of it have disappeared within the anus, and its tip has been felt about the umbilicus, and even in the right iliac region; but the mobile sigmoid flexure has explained it all, and beyond that loop the good tube has not passed. In actual treatment, I think that the passing of the long tube is to be regarded rather as a solemn ceremonial, that diverts for a moment the patient from his woes, and administers a moral balm to the troubled mind of the surgeon.

Dr. Long Fox: At the Bath meeting of the British Medical Association, Mr. Jonathan Hutchinson gave it as his opinion that most cases of intestinal obstruction could be relieved by the surgeon, if only the physician had not touched the cases previously.

It is an evidence of a more sensible feeling, when surgeons, with the courage induced by the use of antiseptics, meet physicians, who have acted on the grand principle of physiological rest, in friendly conference on the question of obstructions of the bowels.

Without going into minute particulars, we have to deal with: 1. Fæcal accumulations. 2. Strictures from various diseases of the bowel itself, especially malignant disease. 3. Intussusception. 4. The various forms of volvulus. 5. Constriction by bands, &c., external to the gut.

1. It need hardly be said that in cases of fæcal accumulation, ileus paralyticus, no operative interference can be expected to do good. He wins who waits. The best therapeutical agent, the great element of time, will, with belladonna and other means that will suggest themselves to every good practitioner, surely conquer all such cases. It is in these, too, that galvanism proves so useful. When, by patience and perseverance, constipation so aggravated that raw peas eaten weeks before had sprouted in the intestine as in a fertile soil, may be overcome, as in a case of Mr. Wickstead, nothing under this category need be despaired of. *

2. In fatal cases of stricture from diseases of the bowel itself, especially malignant disease, there is in my experience always a certain amount of passage found post-mortem. The constriction, that has been going on for a long time, often rather suddenly causes complete obstruction, not because the passage is organically closed, but because the pressure from above on the latest portion of the growth causes spasm. Here, too, patience, opium with a free hand, and only such other interference as will render the fœcal matter liquid, will often prolong life. But as in the more common forms, the malignant, the lesion is in a large majority of cases in the sigmoid flexure or the descending colon, lumbar colotomy renders the life of the patient a shade less miserable than would be the case after operation in front. A patient should always have the offer of this operation made him, and most of us have met with some cases in which not only has life been prolonged, but death rendered more easy by such means. I am, however, disposed to consider that the intestinal passage should always be made to terminate at the false anus.

* Belladonna somewhat excites peristalsis, probably by its paretic effects on the vasomotors of the intestinal vessels. The vascular supply being thus increased, augmented action of the muscular coat results.

3. As to intussusception, there will be great difference of opinion. If the mortality without operation is 70 per cent., and the mortality of operation is 72 per cent., the balance is pretty even. No doubt these cases sometimes recover: no doubt in some the parts become glued together, the intussusceptum is separated by gangrene and passes per anum. But such efforts of nature are frequently but step-mother's kindness, bad at best; and a large proportion of such cases die, even though the intussusceptum is got rid of. Better results may be expected from operation in acute cases than in chronic, and the surgeon may justly say that his results will be far more favourable when cases are submitted at the earliest stage. I profess myself in favour of operative interference; but a certain number of good results occur under the use of opium and almost complete starvation.

4. The various forms of volvulus should from the first be treated surgically; volvulus of the sigmoid flexure, and probably also volvulus of the cæcum and ascending colon, by operation only. But I claim for volvulus of the small intestine the possibility of spontaneous cure (without opening the abdomen) by varying the position. Mr. Ormerod had such a case some years ago, where rubbing the bowels whilst the patient was placed in an inverted position, the head on the ground, removed exceedingly grave symptoms, doubtless due to this form of lesion. I saw with two other medical friends a case that was being nursed by two weak old crones, and with the restlessness of approaching death insisted on being lifted out of bed. They let him fall with great violence on the floor, and he began to recover from that moment. But such cases will be exceptional, and as a rule early operative interference in all varieties of volvulus should be recommended.

5. In constriction by bands, &c., outside the bowel there is no safe course but operation. Of course it is not impossible that, under opium and starvation, means to avoid even the slightest peristalsis, a knuckle of bowel so constricted may make a spontaneous recovery. But even in such cases the same catastrophe may easily recur.

For the safety of the patient, as well as for accurate diagnosis, aperients should be avoided, until you are sure you have only fæcal accumulation without organic change to deal with, and even then they should be used with caution.

The diagnosis is everything, and for aid in this Mr. Treves' book is simply beyond all praise. It is not a year ago that laparotomy was performed in one of the most noted London hospitals, in a case of typhoid fever, under the idea the case was one of intussusception.

But both for purposes of diagnosis, and in considering the question of operation, we should remember that we are dealing with parts profusely supplied by nerves, the chief feature of whose action is that it is reflex. Any operation means of necessity lesion of nerves whose reflex connection with the heart is exceedingly intricate and close. In diagnosis, the symptom of pain is frequently reflex, and its position, therefore, no evidence of the seat of the lesion; the vomiting, too, is, in the early stage of acute cases, a reflex phenomenon, and no absolute evidence of the position of obstructed bowel; and the anuria, though partially dependent upon shock and the consequent diminished pressure of blood in the aorta, is in acute cases not seldom due to a reflex irritation of the renal plexus.

Dr. Shingleton Smith: The subject of intestinal obstruction is of interest to, and is within the province of, both physician and surgeon alike; and as its treatment is usually in the first instance medical, and then afterwards when medicine fails becomes surgical, it is desirable that physician and surgeon both should have certain definite lines of treatment agreed on by all. In consequence of the difficulty in the accurate diagnosis of the causes of internal strangulation, and the absence of a well-defined line of demarcation between it and simple obstruction from retention of fæces, there is a danger on the one hand that a formidable, and commonly fatal, surgical operation may be performed when recovery is possible without anything more than medicinal interference, and on the other hand that medicinal remedies may be given with no other result than waste of time, and surgical aid called in only when too late for a prospect of success.

If the diagnosis of internal strangulation were as clear as it is in cases of strangulated external hernia, there could be no more reason for doubt or delay as to the steps necessary for its relief; but the occurrence of cases in which the characteristic features of obstruction are present with no impassable barrier, and the frequent recovery of such cases without operative interference, are apt to give rise to injurious methods of treatment and fatal delay, in cases where delay is so proverbially dangerous. In consequence of this difficulty, the proposition submitted to the medical section at the Bath meeting of the British Medical Association in 1878 may still be considered *sub judice*, "that, in the present state of surgical knowledge, exploratory operations for the relief of abdominal obstruction, the cause of which cannot be diagnosed, are not warrantable" (Hutchinson).

I propose to deal with some points in the early medicinal treatment of cases of acute obstruction.

The objects of treatment must aim at the removal of the causes, as well as the alleviation of the symptoms; and the most important indication will be to give *rest* to the muscular walls, as well as to the actively secreting mucous membrane of the intestinal tract. All authorities agree that purgatives of all kinds "can do no good, may do immense harm, and must be altogether discarded" (Bristowe); and yet how often is this rule overlooked or wilfully disregarded! The occurrence of abdominal pain, with sickness and constipation, are looked upon as evidences of something needing removal by purgation; and the fact that these are precisely the leading symptoms associated with obstruction is overlooked or forgotten. It cannot be too strongly urged that partial rest—not the absolute rest of paresis—to the intestinal walls is the first and most important indication for treatment; and that purgatives, by stimulating the muscular walls, tend to increase an intussusception, and to increase the vomiting in other forms of obstruction, partly by exciting muscular action, and partly by promoting secretion which can be discharged only by vomiting.

The difficulty as to the administration of aperients clearly arises from the absence of a definite line of division between the symptoms of cases of acute obstruction which require absolute rest, and those of a more chronic form, where a mere increase of the ordinary *vis a tergo* peristaltic action suffices to relieve the constipation, and with it the pain. If it could be satisfactorily diagnosed that an impassable obstruction existed, then purgatives would of course be withheld; but, in the absence of the data for such conclusion, the use of purgatives is adopted, partly as a method of treatment of the patient, and partly in aid of the diagnosis. In many cases the establishment of the existence of an impassable obstruction is an inference from the failure of all aperients to do more than aggravate the pain and vomiting.

Pain is usually the symptom which calls most urgently for relief, and hence the almost universal practice of giving opium where obstruction is suspected. This drug, by its power of arresting peristalsis, by checking secretion, and by its anodyne properties, appears to be the one thing needful. It might, however, be given hypodermically more frequently than it is; and its use in this way, or in suppository, is more especially indicated in cases of obstruction, where vomiting is frequent and where little absorption is going on through the mucous membrane of the stomach and small intestine.

There are, however, two important drawbacks to its use: the one, that it keeps up the constipation; and the other, that it arrests pain and vomiting without necessarily removing the cause on which the pain, vomiting, and constipation depend. These disadvantages have led to the employment of belladonna, either alone or in combination with opium; but authorities differ remarkably as to the mode of action, the dose, and the effects of this drug on the alimentary canal. A recent writer (Dr. Macleod) states that belladonna is now comparatively little used, and that atropia is little heard of in our day (*British Medical Journal*, 1876, vol. ii., p. 673). Mr. Treves, in his recent *Jacksonian Essay*, ignores it altogether. On the other hand, most authorities write favourably of its use, and some are enthusiastic in its favour. The experience of Dr. Norman Kerr (related in the *British Medical Journal*, 1878, v. ii., p. 307) is quite in accord with that of many others, who find that the administration of doses of two grains (or less) of the extract every hour speedily gives relief in cases which at first seem to be hopeless.

As regards the physiological action of belladonna on the intestinal canal, various authors do not agree. Foster (*Text-book of Physiology*) classes atropia amongst the drugs which induce strong peristaltic action. Landois (*Text-book of Human Physiology*) mentions belladonna as a drug which diminishes the excitability of the plexus myentericus, *i.e.* which lessens or even abolishes intestinal peristalsis. Mitchell Bruce (*Mat. Med. and Therapeutics*) has the following passage: "The inhibitory branches of the splanchnics in the intestinal walls are depressed by atropia, which thus increases the peristaltic movements, and causes relaxation of the bowels." Sidney Ringer (*Hand-book of Therapeutics*), in alluding to the assertion that belladonna increases the peristaltic movement, mentions the proof that it paralyses the terminations of the inhibitory fibres of the splanchnics : "thus stimulation of the splanchnics will stop intestinal movement, but small doses of atropia will prevent this arrest."

The observations of physiologists appear, therefore, to be somewhat conflicting as to the sum total of the effects of belladonna on the peristaltic action of the bowel ; but, although it appears to be established that the drug does diminish the excitability of the plexus myentericus and paralyses the inhibitory action of the splanchnics, it has not been proved that the motor fibres from the vagi are in any way affected, so far as the intestine is concerned. Accordingly, it may be inferred that, the inhibitory action of the splanchnics being in abeyance, the excitor action of the pneumogastrics is allowed to have full play.

Although the physiological evidence is still incomplete, there can be no doubt of the fact that the whole effect of the drug on the peristaltic action of the bowel is not that of paralysis, and hence the general consensus of opinion that the practice advocated by Trousseau, of giving belladonna in obstinate constipation is a good one which should be generally adopted. This being the case, the question naturally arises whether, in those cases of intestinal obstruction in which a definite diagnosis is not possible, the anodyne effects of belladonna, which will not constipate, will not be more satisfactory than those of opium, which does tend to constipate. Most authorities recommend the administration of both drugs at the same time. Hutchinson's rule (*British Medical Journal*, 1878, v. ii., p. 306) is as follows: "Opium (or morphia) must be used in proportion to the pain which the patient suffers. It should be administered by the rectum or hypodermically, and should be combined with belladonna. If there be not much pain or shock, it is better avoided, since it increases constipation and may mask the symptoms." The latter part of this rule applies to opium, but need not apply to belladonna, since the latter drug neither increases the constipation nor masks the symptoms. The feeble anodyne properties of belladonna are its principal drawback; but for this, and opium might be discarded altogether. It seems desirable that, should opium be necessary, its administration should be by hypodermic injection, in order that its local effects in causing constipation may be less pronounced. In one other respect belladonna has the advantage over its rival, opium: its well-known influence in arresting secretion—easily proved with regard to the skin and the mouth, but not absolutely established as regards the other secretions of the alimentary canal—enable it to favour intestinal rest so far as the mucous membrane is concerned. The total effects of belladonna appear then to be, a feeble anodyne, a gentle stimulant to the muscular walls, a powerful sedative to the mucous membrane : it will therefore tend to allay pain and arrest secretion, but at same time will not perpetuate the constipation.

It seems necessary that a distinction should be made in the treatment of intussusception and other forms of acute obstruction ; in the former, absolute arrest of peristalsis and a powerful anodyne are both urgently required, and opium in heroic doses will act most efficiently ; in the latter class of cases, belladonna alone would appear to be the most suitable, and opium may be reserved for hypodermic or rectal administration only in case the belladonna should fail to relieve the pain.

The first indications for treatment, relief to pain, arrest of violent peristaltic action, and diminution of secretion, having been accomplished or attempted, some further measures may then be adopted with a view to the removal of the cause of the obstruction. These will comprise enemata of various kinds, and insufflation of air. Ordinary enemata, but in unlimited quantity and in the inverted position of the body, aided also by the long tube, should be frequently repeated.

Other methods of treatment, such as electricity and massage, are commonly advised ; but it should never be forgotten that, if an actual twist or strangulation by band be present, such measures cannot be of any avail, but may be productive of much harm, the former by exciting peristalsis, the latter by causing a possible rupture of the weakened bowel. Treatment of this kind can only be of service in cases of fæcal accumulation with no urgent symptoms of obstruction, and in cases of acute obstruction these methods occupy the time of the physician and the attention of the patient, when both should be directed to more active interference of a surgical character. They can be of no avail when an impassable barrier exists, and they only tend to waste valuable time and impair the prospect of relief from active surgical measures.

One other important element of treatment relates to the diet. Clearly it is worse than useless to attempt to introduce solid food or even liquid nourishment into the stomach whilst vomiting is frequent. If, however, the patient is able to retain any liquids, then the most perfect food will be peptonised milk, which will not give rise to any gastric irritation from the formation of curd, and may be absorbed from the upper part of the intestine, leaving little residue comparable to the 25 per cent. of dyspeptone from ordinary milk. The difficulty associated with the preparation of peptonised milk, and of maintaining the supply, is now surmounted, inasmuch as a new condensed peptonised milk has been prepared by Messrs. Savory and Moore, and is sold in tins. This can be given in a more or less concentrated form, and will supply a most valuable nutriment, already digested, and in a convenient form always ready for use, pleasant to the palate, and not liable to the bitterness arising from over digestion, as when the milk is peptonised in the ordinary way.

Should the patient be unable to retain even peptonised milk when given by the mouth, then feeding by the rectum becomes a necessity, and here the peptonised milk is again of the greatest utility. Various observations have shown how feeble is the digestive power of the rectum, and that accordingly for rectal alimentation previously prepared peptone must be superior to the various other substances usually employed in enemata.

The absorptive power of the rectum is no doubt great, and it has been shown that fluid egg albumen, milk and its proteids, flesh juice, and other substances may be absorbed; yet the osmotic properties of previously prepared peptone, together with the fact that it leaves no residue behind it, give peptonised milk manifest advantages over all other kinds of food for rectal administration. Two pints of peptonised milk daily are usually sufficient to maintain the weight of an average patient when in a state of complete rest; and there can be no difficulty, in cases of chronic obstruction of the bowels, in counteracting the tendency to death by exhaustion whilst other measures are being adopted to relieve the obstruction.

The questions, when to operate, and what operation is the best, can only be decided when the details of the individual case are being considered; but my own experience would lead me to ask for surgical assistance in any case where symptoms of acute internal strangulation were not relieved by medicinal treatment in a period of about forty-eight hours.

Mr. Nelson C. Dobson: The subject of intestinal obstruction is so varied and extensive, that it would be impossible in a short time to give even an outline of its various forms, with their symptoms, diagnosis, and treatment. This is a task I do not intend to attempt; but feeling that most of us have formed some ideas on some at least of the points under discussion, either from careful reflection and earnest thought, or practical experience, or a combination of all these circumstances, it occurred to me that I could best take my small share in the evening's work by taking up one branch of the question. I have, therefore, being led by inclination, and the force of circumstances, selected as the basis of a few observations the part of the subject which refers more especially to *acute* intestinal obstruction, particularly in its surgical aspects.

In speaking of acute intestinal obstruction, I include all those cases in which the ordinary symptoms of acute obstruction manifest themselves in a patient who has hitherto been free from intestinal troubles, and in whom no external hernia is discoverable; and it goes without saying that in the remarks which follow I exclude cases in which the cause of occlusion is a slowly forming stricture, whether simple or malignant, of any part of the intestinal tract, or any form of chronic obstruction. The symptoms and treatment of this latter class are so well recognised and understood, that I consider any further comment at the present unnecessary.

Now it is known that if we exclude volvulus, which may occur in either the large or small intestine, though it occurs much more frequently in the large than the small, that most of the cases of acute intestinal obstruction have their seat in the small intestine: this may help us a step forward.

In a general sense, and including, without going into details, the acute obstructions of both large and small intestines, we may say that they may be classified as being due to either:

1. Strangulations by bands of various kinds, or internal hernias from loops of intestine slipping through apertures in the mesentery or omentum.

2. Volvulus either of large or small intestine.

3. Acute intussusception of either large or small intestine.

4. Obstruction by foreign bodies, either within the bowel, or tumours from the outside pressing upon and occluding the lumen of the bowel.

It is known that tumours in this situation may, like some forms of stricture of the intestine, progress unobtrusively up to a certain point, and then suddenly give rise to symptoms characteristic of acute obstruction.

The mere recital of these forms of obstruction might almost lead one to suppose that the question of determining the precise cause of occlusion in any given case might be a simple matter; but in actual practice it is by no means easy. There is much yet to be learnt and perfected in the way of diagnosis before the surgeon can hope satisfactorily and scientifically to relieve these cases by operation. I look to careful work and accurate record of details to accomplish much in the future.

I have no intention myself to attempt anything like a differential diagnosis; I am conscious that, in the present state of our knowledge, the most we can do in many cases is to determine the actual existence of acute intestinal obstruction, and in some instances perhaps its seat; but in most the precise anatomical lesion that determines the occlusion cannot be diagnosed, whether it be by bands, or acute kinking, or other cause. It is true that certain well-marked conditions—such, for example, as ileo-cæcal or colon invaginations—usually present such symptoms as lead to a diagnosis; but the same condition occurring high up in the ileum may defy diagnosis previous to operation; and I should be strongly inclined to doubt the diagnosis of such cases as "volvulus of small intestine" when such diagnosis has not been verified by operation or post-mortem examination.

We may occasionally meet with difficulties of another kind; viz., the existence of symptoms leading us to suppose that mechanical obstruction of an acute character exists, when in reality there is no such condition present, as in some forms of peritonitis, and especially perforative peritonitis, associated with ulceration of the appendix or cæcum (not from disease), but from the presence of foreign bodies, such as plum or cherry stones; of this a remarkable instance came under my notice some years ago. A middle-aged man, previously healthy, suddenly developed the symptoms of acute intestinal obstruction, with constipation and fæcal vomiting, and died in four days. After death no mechanical obstruction was found; but two plum-stones were discovered, which had ulcerated through the cæcum near the appendix, and these, with the fæcal extravasation which followed, produced the fatal peritonitis, which so closely resembled in the symptoms a case of acute obstruction.

Had this patient come under my care to-day, I should think it very likely he would have been the subject of a laparotomy, and though I should have found no mechanical obstruction, I should not altogether have regretted the operation; indeed, I should, with a full knowledge of the condition, have advocated abdominal section on the same principle that I recently advocated that operation in perforating ulcer of the stomach; viz., dealing with the perforation in such manner as circumstances might indicate, combined with a thorough cleaning up of the peritoneal cavity. Of course I am aware that, for success to attend such a procedure very early interference would be necessary, and here again the question of diagnosis would be the obstacle.

Opponents of laparotomy have advanced the fact that I have just mentioned (that of finding no mechanical obstruction, though the ordinary signs of such a condition have been closely simulated) as one of the reasons against the operation; but even supposing that perforative or non-perforative peritonitis may be occasionally mistaken for obstruction, I still think that abdominal section might sometimes be beneficial in these cases.

Again, the opponents of operation say that the intestine may be ruptured in the search for the obstruction, and that this accident has actually happened to such surgeons as Billroth and Fergusson. They also say that many surgeons have failed to find the obstruction, on account of the numerous adhesions binding the bowels together; and still further, and with much truth, they assert that in volvulus, for example, the twist is often so extraordinarily complicated that it can only be unravelled even in the dead body by a careful study of the specimen. With regard to volvulus, I am prepared to admit that most surgeons agree it is not a condition that can generally be relieved by laparotomy.

If we appeal to statistics we have not much to encourage us; Leichtenstern gives 79 cases of laparotomy for obstruction, with 55 deaths or 70 per cent.; but as successful cases have a better chance of being reported than unsuccessful ones, probably even these statistics are too favourable of the operation as it has hitherto been performed.

Notwithstanding all this, I cannot help thinking, that with more refined and accurate diagnosis and earlier interference, combined with the recent advances in abdominal surgery, we may expect better results in the future than have been obtained in the past. I therefore strongly advise operation, where medicinal means, such as diet, quiet, and opium, have been fairly tried for twenty-four hours, and the symptoms growing worse, and especially in those cases in which we may be able to eliminate volvulus or acute intussusception as the cause of the acute obstruction. It seems to be pretty well recognised that strangulations by false bands and internal hernias offer the best field for operation.

Having decided to operate, we have further to decide the particular operation that shall be undertaken, and the details of its performance. Briefly it may be said we have puncture of the intestine by a small trocar for the relief of meteorism; laparo-enterotomy (Nélaton's operation), the establishment of an artificial anus without search after the seat or nature of the obstruction; and laparotomy, for the purpose of dividing bands or liberating otherwise the incarcerated bowel: and this operation may be combined or not with enterotomy or enterectomy, the latter operation being necessary for gangrenous intestine or irreducible intussusception; the latest statistics of this operation, recently quoted by Mr. Whitehead, seem to point to the advisability of establishing an artificial anus in the first instance, and subsequently restoring the lumen of the intestine by operation, rather than attempting to restore the continuity of the bowel in the first instance.

The plan advocated by Mr. Treves of performing laparotomy seems to me on the whole to be the best one; viz., that of making a median incision below the umbilicus sufficiently large to admit the hand, and directing one's search in the first place to the cæcum, and then following up the ileum from this point. It is advisable, if possible, to prevent the extrusion of the distended intestines. I have experienced difficulty in returning into the abdominal cavity distended coils which have thus become extruded.

I have no doubt that improvements in abdominal

4

surgery will lead to more perfect methods of treating acute intestinal obstruction by operative means; but I am not sanguine that this field of abdominal surgery will produce brilliant results. I do, however, feel certain that by steady, honest work, and careful records of cases, a more precise diagnosis is possible, and as a consequence that laparotomy for acute intestinal obstruction will be established on a firmer basis in the near future than in the past.

Mr. Cross: Mr. President, I shall confine my remarks to cases of acute intestinal obstruction. The symptoms resemble those found in ordinary strangulated hernia—sudden onset of pain in the belly, with general nervous shock, accompanied by vomiting and constipation—and depend on some constriction of the wall of the intestine, of its vessels and nerves, with occlusion of its canal.

No rule in surgery is more absolute than that immediate relief should be given to the strangulated bowel of external hernia; and when operation is necessary for division of the strangulating band, the prognosis mainly depends on the length of time during which the constriction of the gut has existed.

In a quarter of the cases of all kinds of intestinal obstruction, the circumstances of the bowel are precisely similar to those of ordinary hernia; it is pressed on by a band or adhesion of some kind, or is locked in a slit of some part of the peritoneum. Such cases imperatively demand immediate relief by laparotomy and division of the constricting tissues.

Intussusception is a still more common cause of intestinal obstruction; if enemata and abdominal taxis fail to produce any good result, there should be no hesitation about applying intra-abdominal manipulation; and this procedure should be resorted to in volvulus, and in acute obstruction by gall-stones or other foreign body. I do not say that the diagnosis can be absolute before operating; but by careful consideration of all the facts of the case a very strong presumption as to the kind of lesion may be arrived at, upon which an intelligent exploration of the belly can be carried out.

The past history of the case is all important; acute symptoms may supervene on any form of chronic obstruction. Peritonitis produces false bands and adhesions, especially where it covers the pelvic organs of the female, and close to the cæcum of the male. Hernia lengthens the mesentery and omentum, and causes peritonitis. Attacks of colic and ill-health precede intussusception, which in half the cases is accompanied by a tumour felt along the large bowel during a paroxysm of pain, and by diarrhœa with blood instead of constipation and vomiting. Volvulus, most usual at the sigmoid flexure, produces much meteorism and dyspnœa by pressure on the diaphragm. Intussusception is most common in children; bands between the ages of twenty and forty; and volvulus in later life. The physician, with his highly educated powers for diagnosis of deep-seated organs, may feel sure of the conditions of the case; no surgical interference is admissible, or it may be imperative.

In uncertain cases, at any rate with acute symptoms, an early exploratory operation should be done. This is a thoroughly recognised procedure in other fields of abdominal surgery. It allows of a definite diagnosis being made, and in *competent hands*, where no further surgical interference proves necessary, it scarcely adds more to the patient's danger than the use of opium and belladonna. In very many cases it will indicate that further surgical interference is the only direction from which help can come. Our present difficulty lies in diagnosis; as this becomes more accurate, surgery will acquire a greater share in the treatment of these cases.

Dr. E. Markham Skerritt: The difficulties which arise with regard to the treatment of intestinal obstruction are all dependent upon difficulties in diagnosis. Given the exact anatomical condition existing, the decision as to the treatment appropriate to it is easy. Cases are now and again met with where all the symptoms point to acute internal strangulation of the bowel, and yet recovery takes place without operation, and where consequently it is highly probable that the supposed condition did not exist. Add to these the frequent instances in which, with a similar clinical history, operative interference or post mortem examination reveals, not a mechanical strangulation, but a condition not admitting of relief by surgical measures, and it becomes evident that in many cases an accurate diagnosis cannot be arrived at, and that a corresponding degree of uncertainty must surround the all-important question of treatment.

It must, however, be clearly borne in mind that, if internal strangulation exists, there is no more probability of relief apart from operation than there is in the case of an external hernia; and that consequently in the former case the practitioner is no more justified in relying upon rest, and diet, and drugs, than he would be in the latter. The question, therefore, arises: In a case where a careful examination of the evidence affords a reasonable presumption that acute internal strangulation exists, is it justifiable to undertake what must be more or less an exploratory operation? Looking to the inevitable result if such a strangulation is present and remains unrelieved, and having regard also to the probably fatal termination in any case of a condition where the symptoms of acute strangulation depend upon a non-mechanical cause, this question must be answered in the affirmative. If, therefore, acute internal strangulation is believed to exist, the treatment adopted should be similar to that followed with an external hernia. First, abdominal taxis, so highly spoken of by Mr. Hutchinson (*British Medical Journal*, 1878, ii. 306), should be employed, sometimes repeatedly, the abdomen being carefully kneaded while the patient is fully under the influence of ether or chloroform, and copious enemata being administered both in the usual way and also in the inverted position of the body; opium and belladonna should be administered, and food should be given only by the bowel. If, in spite of these measures, the symptoms remain unrelieved, the abdomen should be opened and search should be made for the cause of the obstruction; and it is too often lost sight of that, to be of any use, operation must not be too long delayed.

I will briefly refer to a source of fallacy in the diagnosis of intestinal obstruction which I believe is not universally recognised, and which I discussed in a paper read before the Clinical Society of London in 1879 (*Clinical Society's Transactions*, vol. 12, p. 224). It is well known that one of the commonest and most reliable signs of ascites is dulness in the dependent parts of the abdomen, changing its site with alterations in the position of the patient. In cases of intestinal obstruction this physical sign may be present; there may be dulness in either flank when the patient lies on his back, giving place to resonance when he is turned on the side, although there is no free fluid in the peritoneal cavity. This phenomenon is to be explained as follows: Great distension of the intestine exists in such a case; the gut contains, in addition to gas, a large proportion of liquid fæces; and the gas and the fluid in each individual coil of intestine necessarily obey the same physical laws as do the gas-containing intestines and the free fluid in ordinary ascites; *i.e.*, in each coil of intestine the gas will rise to the top in whatever position the patient lies, and the fæces will sink to the bottom. In the diagnosis between acute strangulation and those acute inflammatory conditions in the abdomen which may be mistaken for it, it is evidently important to remember that a physical sign which is commonly supposed to be diagnostic of the existence of free fluid in the peritoneal cavity may be dependent solely upon distension of the intestine.

Mr. Michell Clarke, after some remarks on the relative position of physicians, surgeons, and general practitioners with regard to cases of intestinal obstruction, said: He agreed in the main with what Dr. Markham Skerritt had just said; viz., that the difficulty was one of diagnosis. It was frequently if not generally impossible to make a correct diagnosis early. If this could be done, there were undoubtedly certain cases in which an operation should be done early; but as this was generally impossible, there could be no doubt that in the majority of instances the patient would be treated for a longer or shorter period with medicines, &c.

He did not consider that the responsibility of making a diagnosis so as to establish, or the contrary, the necessity for gastrotomy lay wholly with the physicians, as Mr. Greig Smith seemed to infer, but that it belonged equally to the surgeon who would perform the operation.

Nor did he think that Dr. Smith's proposal that a conclave of twenty physicians and twenty surgeons to discuss the present position of this question would advance it. Rather, it was probable that the diagnosis would at some time or other be made easier by the discovery by some man of genius (he might even now be present in the room) of some means by which it might be made more accurate, as Laennec's discoveries had so wonderfully made clear the different diseases of the thoracic viscera.

It was nearly certain that in the early stages an aperient would be given, and this whether physician, surgeon, or general practitioner should have the first charge of the case. It was absolutely necessary in many cases to ask the question, Is there a way through with an aperient? and in not a few the response would be favourable.

But, of course, if a diagnosis could be made that there was internal strangulation, or volvulus, or intussusception, or, in fact, if it could be made clear that there was an insuperable stoppage of whatever kind, there could be no good in giving aperients. Nor would it be worth while to discuss the question of belladonna, which Mr. Greig Smith considered the only medicine worth thinking about, nor of feeding the patient upon peptonised milk, &c., nor of enemata, nor indeed of anything else; an operation would alone give promise of cure.

He was very glad to hear that an opinion he had long held with regard to the long tube (O'Beirne's) was shared by so high an authority as Mr. Treves. It was well known to the members of this branch that he (Mr. Clarke) had long held that it was very difficult, if not generally impossible, to pass this tube through the sigmoid flexure of the colon. Many years ago he asserted this, on the occasion of the late Mr. Green having a number of cases that he considered he had treated successfully by enemata administered by the long tube, which had been passed beyond the sigmoid flexure of the colon. Subsequently, some experiments were made in the p.m. room of the Bristol Infirmary, and it was found that the tube could not be passed. There was one point, however, that was important, and that had been overlooked ; it was this : The long tube, although it cannot well be made to traverse the sigmoid flexure of the colon, can be passed well into the lower end of it, and in that way enemata can be sent into the intestines that would with the ordinary tube merely enter the rectum and come out again. With the long tube used in this way great good would sometimes be done by letting the fluid flow in by gravitation, the pelvis being well raised, and the reservoir of fluid hung to the top of the bedstead. In this way, after several days of obstruction, he had succeeded in relieving an aged patient of about 90, some two years ago, and this plan of administering enemata was now being frequently adopted.

He considered that the subject of intestinal obstruction was too large for one evening's discussion, and that the further consideration of it might well be adjourned to another evening ; and that it would be well to treat each variety, and especially such varieties as internal strangulation, volvulus, intussusception, &c.; impaction of fæces, or gall-stones, &c. ; pressure from tumours, &c., within or without, &c., upon its own merits.

Dr. Goodridge (Bath) : I will not, Sir, at this hour touch upon any of the topics that have been so fully and so ably dealt with by previous speakers; but there is just one point upon which I should like to be permitted to say a word, as, although it relates more to chronic cases, and particularly to those of obstruction of the lower bowel, it should not pass unnoticed, I think, in a discussion on the treatment of intestinal obstruction. We all know that, in the cases to which I refer, a stage sooner or later arrives when it is not so much acute pain, severe as this is apt to be on occasions of temporary increase of blockage, as the great tympanitic distension of the abdomen, which distresses the patient. It may become extreme, and is mainly due, I need not say, to atony of the muscular fibre of the gut. For the relief of this condition, of late years puncture with needles, or fine capillary trocars, has been proposed; but I have seen very striking results ensue from the judicious use of strychnia. In one case I well remember, of which I had the charge many years ago, before operative measures were as much in vogue as they are at present, the patient was in the greatest possible distress. He had extreme tympanitic distension of abdomen, his diaphragm was pushed up, and his breathing oppressed to a formidable degree; in short, he was in a most critical condition, when, upon the administration of strychnia, followed up regularly for eight or ten days, the case gradually assumed a quite different aspect; the abdomen was reduced to a concave, the breathing became easy, the bowels were largely relieved of their accumulated contents, and the general condition of the patient was improved so much that, with a *pro re natâ* recurrence to the same remedy, in conjunction of course with other appropriate treatment, nearly twelve months, I have no hesitation in saying, were added to his life. The presence of annular stricture of the sigmoid flexure, I should not omit to mention, was proved post mortem. It has occurred to me to witness similar results in other cases.

As, therefore, extreme abdominal distension does not necessarily denote extreme constriction of the bowel, I think that a cautious use of strychnia in any case in which it had not already been used and failed (and fail in every such case of course sooner, or later, it must) should precede the introduction of needles, &c., into the bowel, useful as this measure may be in its turn; lumbar colotomy always remaining as the ultimate resource.

Mr. Greig Smith replied in summary and criticism. He congratulated the meeting upon having given expression to an opinion upon the whole more advanced, and, as he believed, more sound than any that had yet appeared as emanating from a discussion. He had no doubt but that it would have fruitful results in their midst.

